# Can Mini-Dose Glucagon Prevent Fasting-Induced Hypoglycemia and Preserve Fasting?

**DOI:** 10.7759/cureus.98620

**Published:** 2025-12-07

**Authors:** Metab Algeffari

**Affiliations:** 1 Department of Family and Community Medicine, College of Medicine, Qassim University, Buraydah, SAU

**Keywords:** diabetes, fasting, glucagon, hypoglycemia, mini-dose, ramadan

## Abstract

Individuals with diabetes, particularly those using insulin, face substantial challenges when fasting during the Islamic month of Ramadan due to prolonged periods without food or drink and an increased risk of hypoglycemia. Mini-dose glucagon (MDG) has been considered as an alternative to oral carbohydrate intake for mild or impending hypoglycemia without breaking the fast; however, current evidence is limited and remains preliminary, derived largely from small pilot studies and limited real-world data.

This hypothesis-generating review synthesizes findings from a pilot RCT (n=17), an observational survey (n=136), and a focused literature search, providing the reader with the best evidence so far on this topic. Across the available evidence, MDG appears feasible for short-term correction of mild hypoglycemia, yet its efficacy, safety, and optimal dosing during fasting remain insufficiently established. Limitations of the present studies, such as small sample sizes, investigators' involvement, and heterogeneity in study designs, suggest cautious interpretation.

Considering the relatively early nature of the evidence and the importance of patient safety at fasting times, the use of MDG cannot be regarded as a proven or routine strategy. Larger, independently conducted studies are required to clarify its clinical role, validate dosing protocols, and ensure alignment with safety-first recommendations for fasting individuals with diabetes.

## Introduction and background

Hypoglycemia is the most frequent complication of insulin treatment in diabetes and is currently the major obstacle to achieving near normal glucose concentrations [1,2]. It has been reported that 5-10% of children and adults with type 1 diabetes (T1D) experience a severe hypoglycemic episode every year, which is usually associated with noticeable neurocognitive dysfunction, coma, or seizures [2,3]. In addition, repeated hypoglycemic episodes are a risk factor for the development of hypoglycemic unawareness, a condition that greatly increases the chances of severe hypoglycemia [1,2]. This life-threatening state can often lead to costly emergency medical treatment or admission to acute care facilities [4]. In addition to its physical health consequences, severe hypoglycemia has a severe negative effect on the psychological and emotional well-being of patients and their families and thus affects their quality of life adversely [5,6]. Despite advancements in insulin formulations and delivery devices, the risk of hypoglycemia remains a significant concern, contributing to both the acute and long-term complications of diabetes [2-7].

In normal physiology, glucagon plays an important role in regulating blood glucose levels to prevent hypoglycemia by acting as a counterregulatory hormone, raising blood glucose levels to prevent low blood sugar. During fasting, this delicate insulin-glucagon balance becomes increasingly dependent on hepatic responsiveness and insulin sensitivity. As a result of T1D, however, this defense mechanism is impaired at an early stage [8-10], and the combination of relative insulin deficiency and variable insulin resistance, particularly in those using exogenous insulin, disrupts normal hepatic glucose release and increases susceptibility to hypoglycemia. Moreover, it has been shown that mild hypoglycemia can often be treated with the consumption of simple carbohydrates, but frequent reliance on this approach can result in unwanted weight gain. In addition, consuming carbohydrate orally, even though it is effective, is not an acceptable option for individuals fasting during Ramadan, as it would break their fast and conflict with their religious practices [11].

At present, the only alternative to oral carbohydrate intake for medically treating hypoglycemia is glucagon therapy. Yet, for many years, this injectable hormone has mainly served to rescue people with diabetes when they experience severe hypoglycemia [5]. In order to use emergency glucagon kits, the glucagon powder must be reconstituted first. Once reconstituted, the liquid is only stable for a short period of time. Due to these limitations, glucagon has not been widely used beyond emergency situations, highlighting the need for more stable and practical formulations [9,12].

Since traditional glucagon therapy has limitations, and fasting during Ramadan presents challenges, developing new formulations and delivery systems for glucagon has gained increasing interest. Hypoglycemia can be managed more effectively, reliably, and in a user-friendly manner with these innovations. One promising approach involves the use of mini-dose glucagon (MDG), which may offer a safe and effective method to treat and prevent non-severe hypoglycemia in individuals with T1D [13]. This strategy could serve as a practical solution for treating and preventing hypoglycemia without breaking the fast, offering a way to address the unique challenges faced by fasting individuals with diabetes.

This review does not constitute a formal systematic review or meta-analysis, but rather a hypothesis-generating synthesis designed to integrate early clinical evidence with contemporary literature to inform future directions of research.

## Review

Hypoglycemia in Ramadan

Fasting during the Islamic holy month of Ramadan requires abstinence from food and drink from dawn to sunset. Although individuals who are ill are exempt, as stated in the Holy Quran (Al-Baqarah, 183-185), many patients with diabetes still choose to fast due to religious, cultural, and social reasons [14]. Nevertheless, fasting poses a significant challenge to maintaining glycemic control, particularly among patients with T1D, who are at greater risk of acute complications, such as diabetic ketoacidosis and severe hypoglycemia [15]. In order to reduce these risks, it is essential to address the unique needs of patients observing Ramadan, since prolonged fasting without calorie intake or hydration can exacerbate the risks [14-16].

The effects of Ramadan fasting on glycemic control and acute complications in diabetics have been studied extensively, consistently highlighting an increased risk of hypoglycemia, particularly in T1D. As one study found, up to 18% of patients with T1D had at least one episode of hypoglycemia during Ramadan, but most kept fasting despite it [17]. Another study revealed that 75% of participants avoided breaking their fast near Iftar (the evening meal) even when experiencing hypoglycemia, and the same percentage avoided treating mild hypoglycemia during fasting hours as well [18]. Furthermore, research has reported that the prevalence of hypoglycemia and hyperglycemia among patients with T1D during Ramadan was 36% and 58%, respectively. In patients with type 2 diabetes (T2D), severe hypoglycemia during fasting occurs 6-9% of the time [17,19]. Fasting increases the risk of hypoglycemia for a variety of reasons, including altered eating patterns, delayed meal timings, and medication adjustment. It is evident from these findings that managing diabetes during Ramadan is a significant challenge, and that more effective strategies are needed to ensure patient safety [19].

Managing diabetes during Ramadan fasting

Many organizations including the American Diabetes Association (ADA) and the International Diabetes Federation in collaboration with the Diabetes and Ramadan Alliance (IDF-DAR), have developed guidelines and recommendations to address the challenges associated with fasting during Ramadan for patients with diabetes. In order to improve glycemic control and minimize the risk of acute complications during Ramadan, these guidelines emphasize that pre-Ramadan assessments, education, and medication adjustments are crucial [20-22].

According to the recommendations, patients with diabetes should be classified into risk groups based on factors such as diabetes type, glycemic control, complications, and a history of hypoglycemia to determine how high, moderate, and low risk they are. In general, patients at high risk are advised to refrain from fasting, while those at moderate risk can fast under close medical supervision and with structured education [20,22].

As a result, the guidelines recommend that for patients who choose to fast, medication regimens be modified to comply with fasting patterns, such as reducing doses of insulin or oral antidiabetic medications, and adjusting their timing to align with fasting and non-fasting periods. Despite this, the current guidelines do not address the issue of strategies to maintain the fast while timely management of hypoglycemia [21]. One emerging approach is the use of MDG, which has shown promise in treating and preventing non-severe hypoglycemia in individuals with T1D without requiring the fast to be broken. In addition, this innovative strategy offers a practical and culturally sensitive approach to managing hypoglycemia that addresses a critical gap in care for fasting individuals with diabetes, paving the way for safer fasting practices for all. Moreover, there is still much research to be carried out to determine which medication strategies work best during Ramadan fasting. 

To summarize the most relevant international recommendations, Table 1 presents a comparative overview of key guidance from the IDF-DAR (2021) and ADA (2024) guidelines, including aspects of risk stratification, pre-Ramadan assessment, and medication adjustments [23,24].

**Table 1:** Summary of the key international recommendations for safe fasting during Ramadan IDF-DAR: International Diabetes Federation in collaboration with the Diabetes and Ramadan Alliance; ADA: American Diabetes Association

Domain	IDF-DAR 2021	ADA 2024
Risk stratification	Structured 3-tier risk model (very high, high, moderate/low). Fasting discouraged for very high-risk individuals.	No Ramadan-specific risk tool; individualized assessment based on comorbidities and hypoglycemia risk.
Pre-Ramadan assessment	Evaluation 6–8 weeks prior, with medication review, risk assessment, and structured education.	Pre-fasting counseling with individualized glycemic targets and medication adjustments.
Medication adjustments	Detailed, Ramadan-specific dose adjustment guidance for insulin and oral agents.	General adjustment principles; no Ramadan-specific algorithms.
When to break the fast	Stop fasting if glucose <70 mg/dL, >300 mg/dL, or with symptoms of illness or dehydration.	Stop fasting with symptomatic hypoglycemia, dehydration, or significant hyperglycemia.
Education & lifestyle	Structured Ramadan-focused education on meals, hydration, sleep, and activity.	Emphasis on diabetes self-management education, meal planning, and activity adjustment.

This comparative summary aims to enhance clarity and clinical applicability for healthcare professionals managing diabetes during Ramadan.

Glucagon physiology and action

In 1923, the glucagon protein, discovered by Murlin et al., was identified as a component of pancreatic extracts that caused transient hyperglycemia in rabbits [25,26]. The glucagon hormone is a 29 amino acid peptide hormone produced by the pancreatic islets. As a counter-regulatory hormone to insulin in order to maintain blood glucose levels within a narrow physiological range, it plays a crucial role in glucose homeostasis [27].

A primary function of glucagon is to stimulate the production of hepatic glucose by promoting glycogenolysis (the breakdown of glucose from glycogen) and gluconeogenesis (the formation of glucose from non-carbohydrate precursors). It has been shown that when glucagon binds to its receptor on hepatocytes, it activates adenylyl cyclase, which in turn leads to an increase in intracellular cAMP levels, and ultimately activation of protein kinase A. As a result of this signaling cascade, the glycogen that has been stored in the liver is broken down and glucose is produced from amino acids and lactate as precursors [28].

In addition to inhibiting hepatic glucose production, glucagon also inhibits insulin secretion from pancreatic beta-cells, thereby ensuring that glucose is available during hypoglycemia. Its secretion is tightly regulated by blood glucose levels, with hypoglycemia acting as a potent trigger for glucagon release [26].

MDG exerts its effect primarily by stimulating hepatic glycogenolysis and gluconeogenesis, thereby releasing glucose from liver stores into the circulation. During fasting, insulin levels decline while glucagon secretion increases, maintaining glucose homeostasis through this hepatic mechanism. Administering a small exogenous glucagon dose mimics this physiologic process without significantly altering the fasting state, as it does not require carbohydrate ingestion. The glycemic response to MDG, however, can vary based on several factors, including residual insulin activity (“insulin on board”), hepatic glycogen availability, and the timing of administration relative to fasting duration. For clinicians, understanding this balance between glucagon action and insulin presence is key to optimizing MDG use and anticipating individual variability in glucose response.

Glucagon for hypoglycemia rescue: current formulations

Since the 1970s, glucagon has been used as a cornerstone therapy for severe hypoglycemia outside of emergency medical settings. It remains the only treatment approved by the FDA for those who are unconscious and unable to consume carbohydrates [9]. There are several emergency kits, such as the GlucaGen® HypoKit (Novo Nordisk®) or the Eli Lilly Glucagon Emergency Rescue Kit, that are used to administer glucagon intramuscularly or subcutaneously. However, despite the fact that these kits have proven to be effective, they have significant limitations [29].

Both formulations require reconstitution of lyophilized glucagon powder with a diluent immediately before use, which can make it difficult to administer the medication in a timely manner, especially during an emergency. There are many challenges associated with managing this complexity for caregivers who do not have medical training, especially when they are under stress [29]. A study reveals that only 13% of caregivers were able to administer the full dose of injectable glucagon during a simulated severe hypoglycemia event, proving the practical challenges with these formulations [30]. Due to this, injectable glucagon is often underused and underprescribed, which limits its overall impact as a life-saving treatment. It has been reported that glucagon has a very good safety profile, but there have been many reports that it causes nausea, vomiting, and abdominal pain [9].

As an added complexity, glucagon dosage is weight-based, which means that adults and children over 20 kg are typically prescribed 1 mg, while children under 20 kg are given 0.5 mg or 20 to 30 mcg/kg [31]. However, evidence to support these dosing recommendations is limited. One study indicated that a 1 mg glucagon injection within 30 minutes can increase blood glucose levels by 1.9-3.1 mmol/L, with plasma glucagon concentrations that rise 100- to 200-fold [32]. It has been shown that although glucagon has these effects, the glucose response to glucagon in individuals with insulin-treated diabetes is highly variable, influenced by the levels of insulin and the ratio of insulin to glucagon in the body, along with the timing of the most recent injection.

Addressing limitations and exploring alternatives

In recognition of the limitations of traditional glucagon formulations, ongoing research has focused on developing novel formulations and alternative delivery methods to enhance its usability and efficacy, particularly in emergencies. These innovations can result in reduced handling errors and simplified administration, and patients will be able to achieve more consistent outcomes. A promising solution is ready-to-use glucagon formulations, such as nasal glucagon and pre-mixed auto-injectors, which eliminate the need for reconstitution and improve accessibility during critical situations [30,32-35].

The FDA approved nasal glucagon in 2019 as a needle-free, easy-to-use alternative to injectable glucagon. In 1983, glucagon administered via nasal mucosa was first shown to passively increase blood glucose levels in healthy subjects [32]. This formulation is supplied as a pre-measured, pre-mixed powder that can be administered rapidly by a caregiver or patients themselves by simply inserting the device into the nostril and pressing a button. Studies have shown that nasal glucagon can effectively raise blood glucose levels within 15 minutes, and has a similar safety profile to injectable glucagon [35]. Another emerging option is dasiglucagon, an injectable glucagon formulation that can be administered as a premixed, ready-to-use solution, needing no reconstitution [33]. 

Moreover, the concept of using MDG is gaining traction, especially for treating non-severe hypoglycemia [36]. It is important to note that individuals with T1D can greatly benefit from this approach during Ramadan fasting, or under other circumstances where keeping a fast is a culturally important practice. The use of MDG is a practical and effective method for preventing or treating hypoglycemia without having to break the fast, thus addressing a critical unmet need for fasting individuals living with diabetes.

In this era of ongoing research to refine glucagon formulations and delivery methods, these advances may ultimately lead to a revolutionized approach to hypoglycemia management, allowing patients to be treated safely and effectively in diverse environments.

Methods

This review does not constitute a formal systematic review. Instead, selected Preferred Reporting Items for Systematic Reviews and Meta-Analyses (PRISMA) 2020 principles, specifically the use of a structured search strategy and a transparent study-selection process, were incorporated to enhance clarity and reproducibility, without claiming full PRISMA compliance.

A focused literature search was performed in PubMed, Scopus, and Google Scholar for studies published in English between January 2010 and August 2024. The search strategy used the following keywords and Boolean combinations: “mini-dose glucagon,” “low-dose glucagon,” “hypoglycemia prevention,” “Ramadan fasting,” “type 1 diabetes,” “glucagon analogues,” and “non-severe hypoglycemia.”

Studies were eligible for inclusion if they: (1) were clinical investigations (randomized controlled, crossover, or observational) evaluating MDG in adults or adolescents with diabetes; (2) assessed efficacy, safety, or fasting-related outcomes; and (3) provided accessible full-text data. Exclusion criteria comprised animal or in-vitro studies, non-English publications, and abstract-only reports.

Titles and abstracts were screened for relevance, followed by full-text review of potentially eligible studies. Data were extracted on study design, participant characteristics, intervention protocols, and key outcomes-including glycemic metrics and adverse events. Owing to heterogeneity in study methodologies and outcome measures, the results were synthesized narratively rather than meta-analyzed. This qualitative synthesis highlights the available early clinical evidence and contextual guideline recommendations relevant to Ramadan fasting and the use of MDG in diabetes management.

Mini-dose glucagon (MDG) concept for fasting-induced hypoglycemia

The MDG regimen, first described in 2001, has emerged as a promising strategy for preventing and managing mild to moderate hypoglycemia in both children and adults [36,37]. This approach involves administering smaller, physiologically appropriate doses of glucagon (typically 10-150 µg) to restore blood glucose levels while minimizing the side effects associated with larger rescue doses.

The concept of MDG for fasting-induced hypoglycemia was inspired by its success in managing hypoglycemia during periods of exercise and poor oral intake. In this context, researchers have conducted multiple studies demonstrating that MDG is a practical and effective approach for managing impending hypoglycemia in individuals with T1D, particularly during periods of poor oral intake or during exercise [36,38]. Additionally, the future advancements in insulin pump and artificial pancreas technology will rely on glucagon. Importantly, subcutaneous administration of MDG does not disrupt the fasting state. These factors led to the rationale that a similar MDG approach could provide a viable alternative for managing hypoglycemia during fasting periods, such as the Islamic month of Ramadan, without disrupting the fast.

**Figure 1:**
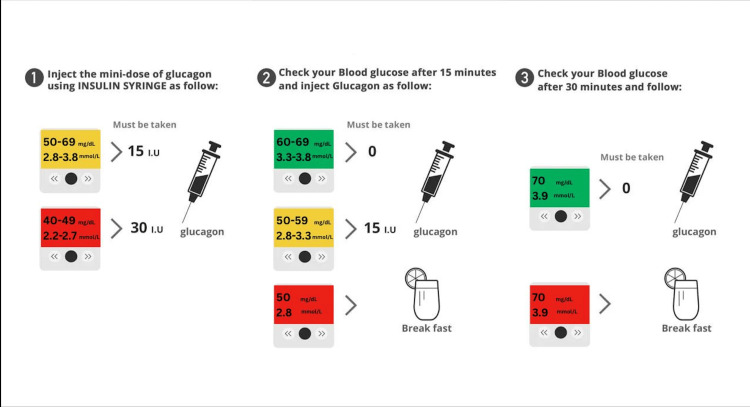
Mini-dose glucagon treatment protocol for fasting-induced hypoglycemia Image credit: The figure was created by the author using Canva (Canva Pty Ltd., Sydney, Australia) [13].

We first introduced the off-label use of subcutaneous MDG in a four-week randomized, controlled crossover study that evaluated its efficacy as an alternative intervention for adults with T1D who fasted for approximately 15 hours daily during Ramadan [13]. Participants wore continuous glucose monitors (CGMs) and were treated with either MDG or oral glucose tablets to correct episodes of fasting-induced hypoglycemia over a two-week period, before crossing over to the alternate intervention for the subsequent two weeks. The treatment protocol involved administering 150 µg of MDG or 15 g of oral glucose for blood glucose levels between 2.8-3.8 mmol/L, and 300 µg of MDG or 30 g of oral glucose for levels between 2.2-2.7 mmol/L (Figure 1).

**Figure 2:**
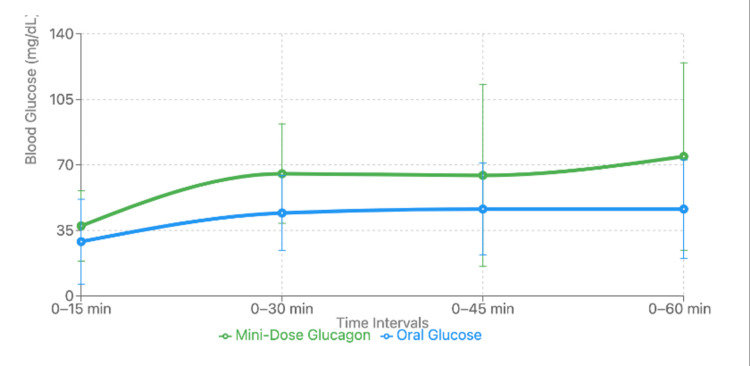
Blood glucose response after using mini-dose glucagon for treating fasting-induced hypoglycemia Image credit: The figure was created by the author using Microsoft Excel (Microsoft Corp., Redmond, WA, USA) [13].

During the study, 80 hypoglycemic episodes were recorded among the 17 participants. Compared to oral glucose, MDG led to significantly greater increases in blood glucose levels at both 30 and 60 minutes after administration (Δt30, P<0.001, Δt60, P=0.02), with these effects sustained even after ≥8 hours of fasting (P=0.01) (Figure 2).

Over the two-week MDG intervention period, participants spent more time within the target glucose range of 3.9-10.0 mmol/L (P=0.009). Furthermore, the use of MDG was associated with a higher rate of successful completion of fasting periods compared to oral glucose (P<0.001). Overall, the study concluded that MDG was an effective alternative for preventing and treating hypoglycemia during fasting, as it improved glycemic control and supported the completion of extended fasts.

Building on these findings, we presented an additional study at the 2024 American Diabetes Association conference, which explored the real-world experiences of people with T1D using MDG [18]. An online survey gathered responses from 136 individuals with diabetes, revealing that 91% of participants were able to successfully complete their fasts after using MDG. Notably, 80% of respondents expressed a preference for MDG over oral glucose for correcting hypoglycemia during fasting periods, reflecting perceived convenience, though it does not constitute evidence of superiority. Moreover, the survey highlighted a significant shift in participants' behavior, with more than half reporting that they now treated even mild or early evening hypoglycemia, which they had previously ignored; however, the clinical significance of this change remains uncertain.

Clinical trials evaluating MDG in adults have consistently reported a favorable safety profile. The most frequently observed adverse effects were mild and transient, including nausea, localized injection-site reactions, and brief episodes of hyperglycemia, all of which resolved spontaneously without intervention. No serious adverse events or treatment-related discontinuations were documented across randomized controlled and observational studies [39,40]. These findings suggest that MDG is a well-tolerated and safe option for the prevention and treatment of non-severe hypoglycemia, including in fasting individuals with T1D. Importantly, the limited sample sizes, short follow-up periods, and the fact that many studies were investigator-initiated restrict the strength of these conclusions.

Beyond clinical trial data, there is increasing real-world experience supporting the off-label use of MDG for prevention and treatment of non-severe hypoglycemia. Post-marketing safety data from both adult and pediatric populations have shown similar tolerability profiles, with no new or unexpected adverse events reported. However, these data are largely derived from off-label use and remain subject to reporting bias. Recent reviews and FDA-approved labeling of novel glucagon formulations (including ready-to-use liquid and nasal preparations) also reinforce the overall safety of glucagon analogues when used in low doses outside of traditional emergency indications [41]. Together, these preliminary findings suggest that MDG may represent a flexible and culturally compatible approach for managing mild hypoglycemia during fasting. However, its safety and effectiveness remain insufficiently established, and existing glucagon formulations have not been formally evaluated for repeated mini-dose use.

These preliminary findings also indicate that MDG may offer a feasible approach for managing mild or impending hypoglycemia during fasting for some individuals with T1D. However, given the small scale of existing studies, their observational nature, and reliance on self-reported outcomes, definitive conclusions regarding efficacy, safety, or superiority over oral glucose cannot be drawn. Larger, independent, and methodologically rigorous trials are needed to determine the appropriate role of MDG in fasting-related diabetes care and to ensure that its use aligns with established safety-first fasting recommendations.

Guideline-based measures and fasting eligibility

The IDF-DAR (2021) and ADA (2024) guidelines emphasize a structured, patient-centered approach to diabetes management during Ramadan [23,24]. Key measures include: 1) Pre-Ramadan evaluation: Conducted six to eight weeks before Ramadan to assess fasting risk, review medications, and individualize care plans; 2) Structured education: Focused on meal planning, hydration, glucose monitoring, and recognition of early warning symptoms; 3) Risk stratification: Classifying individuals into very high, high, moderate, or low risk categories to determine fasting eligibility; 4) Medication adjustment: Modifying insulin or oral agents to coincide with suhoor and iftar; reducing basal doses or using shorter-acting secretagogues; and employing CGM or self-monitoring of blood glucose (SMBG) for real-time titration; 5) Monitoring thresholds: Patients are advised to terminate the fast if blood glucose is <70 mg/dL (3.9 mmol/L), >300 mg/dL (16.7 mmol/L), or if symptoms of hypoglycemia, dehydration, or acute illness occur; 6) Post-Ramadan review: Reassessing metabolic control and adapting long-term therapy based on fasting outcomes.

These measures provide a structured foundation for safe fasting and situate the emerging role of MDG within evidence-based Ramadan care. Consistent with these guidelines, individuals with diabetes should be classified into four risk tiers to guide fasting decisions. The potential role of MDG applies primarily to those in the moderate or selected high-risk groups who choose to fast under close medical supervision. Patients at very high risk should not fast, as the likelihood of acute metabolic complications outweighs potential benefits, even if MDG is available.

The do-not-fast criteria include: 1) Severe hypoglycemia within the past three months; 2) Hypoglycemia unawareness; 3) Recent diabetic ketoacidosis (DKA) or hyperosmolar state; 4) Pregnancy with diabetes; 5) Acute illness or active infection; 6) Brittle or poorly controlled T1D despite optimization

For stable, lower-risk individuals with T1D who are well-educated, adhere to glucose monitoring, and have access to medical support, MDG may serve as a supplementary strategy to treat mild or impending hypoglycemia without breaking the fast. This integrated framework harmonizes MDG use with international recommendations and supports consistent, culturally sensitive fasting safety practices.

Cultural and religious considerations

The use of medical interventions during Ramadan fasting should be approached with both clinical and cultural sensitivity. Islamic scholarly bodies, including the Islamic Fiqh Council of the Muslim World League and Al-Azhar’s Islamic Research Academy, have generally stated that non-nutritive injections do not break the fast (Fatwa No. 93, 1997), as they do not provide caloric intake or nourishment [42,43]. However, some scholars and local fatwa committees have expressed more cautious views, emphasizing that the intent and clinical necessity should be considered on a case-by-case basis.

Accordingly, clinicians are encouraged to discuss such interventions collaboratively with patients, integrating both medical guidance and individual religious perspectives. This approach supports patient autonomy, trust, and culturally sensitive diabetes care during Ramadan.

Limitations and future directions for MDG therapy

While MDG shows encouraging early results, its use remains constrained by several important limitations. Most of the available evidence is derived from small pilot studies, leaving its efficacy, safety, and long-term applicability insufficiently established. To determine its true clinical value, particularly during fasting, larger, well-designed trials with independent replication are needed.

A major practical challenge lies in the currently available glucagon formulations. Traditional emergency glucagon kits require reconstitution of a crystallized powder with diluent, producing a single 1 mg solution intended for the treatment of severe hypoglycemia. Once reconstituted, the solution must be used immediately or refrigerated and discarded within 24 hours, leading to inconvenience and significant waste. Although the 1 mg vial can technically be divided into multiple MDG doses (e.g., six 150 µg aliquots), all remaining portions must still be discarded, making this approach expensive and impractical, especially for individuals who may require repeated MDG during fasting [33].

Newer ready-to-use glucagon products that are stable at room temperature offer improved convenience; however, they are manufactured for full-dose treatment of severe hypoglycemia and are not optimized for mini-dose use. Adapting these formulations for MDG remains costly and operationally difficult. These constraints highlight the unmet need for user-friendly, cost-effective delivery systems specifically designed for MDG. Developing such technologies could substantially improve the management of non-severe hypoglycemia during fasting and help overcome barriers to broader clinical adoption.

The pilot RCT supporting MDG use was limited by its small sample size (n=17), reducing statistical power and generalizability. Recruitment from a single setting may have introduced selection bias, and the crossover design, with direct comparison to oral glucose, may have influenced participant behavior. The accompanying survey also carried inherent limitations, including reliance on self-reported data prone to recall and social-desirability bias, online-only administration that excluded individuals with limited internet access, and the absence of objective validation for HbA1c or glycemic outcomes. Furthermore, variability across study designs, populations, and settings in the reviewed literature restricts the strength of the overall conclusions.

Taken together, the conclusions of this review should be interpreted with caution. Much of the evidence originates from investigator-initiated studies with relatively small samples, introducing the potential for intellectual bias. Additionally, while the review applied PRISMA-inspired methods to enhance transparency, it was not intended to meet the full criteria of a systematic review. Accordingly, the findings are hypothesis-generating and require confirmation through larger, independently conducted trials.

## Conclusions

MDG provides a possible management for mild or imminent hypoglycemia during long-term fasting, such as Ramadan. Preliminary results appear to indicate that MDG may sufficiently increase glucose levels to prevent fasting interruption, although limited evidence is available as it is based primarily on small pilot studies and observational data. As such, its efficacy, safety, and optimal dosing during fasting cannot yet be established with confidence.

While newer, ready-to-use glucagon formulations may eventually enable more practical MDG strategies, their application in mini-dose regimens is still experimental and not specifically designed for fasting contexts. Due to these challenges and our focus on safety-first fasting guidance, MDG should currently be considered an investigational strategy rather than a validated clinical standard.

Robust, independently conducted trials are necessary to clarify the role of MDG in fasting-related hypoglycemia management, define appropriate dosing protocols, and ensure that its use aligns with safe fasting practices for individuals with diabetes.
